# Cancer burden and health inequalities attributable to occupational arsenic exposure: A 32-year global, regional, and national observational study with projections to 2036

**DOI:** 10.1097/MD.0000000000049979

**Published:** 2026-07-31

**Authors:** Di Wu, Xinxiao Li, Minghan Luo, Nian Gao, Xiaowei Wang, Ailin Li, Zhe Mo, Zafar Gazala, Shirui Yan, Lei Wu, Rui Zhang, Junrui Pei

**Affiliations:** a Key Lab of Etiology and Epidemiology, Education Bureau of Heilongjiang Province & Ministry of Health (23618504), Center for Endemic Disease Control, Chinese Center for Disease Control and Prevention, Harbin Medical University, Harbin, Heilongjiang, China; b Public Health College, Qiqihar Medical University, Qiqihar, Heilongjiang, China.

**Keywords:** arsenic, Global Burden of Disease, inequality, machine learning, occupational exposure, prediction

## Abstract

Occupational arsenic exposure (OEA), mainly through inhalation in workplace settings, is an established carcinogenic hazard associated with lung, skin, and bladder cancers. However, long-term trends and the global cancer burden attributable to OEA remain insufficiently characterized. Data from the Global Burden of Disease Study 2021 were used to analyze the cancer burden attributable to OEA from 1990 to 2021. Indicators included summary exposure values (SEVs), deaths, years lived with disability (YLDs), and disability-adjusted life years (DALYs) at global, regional, and national levels. Estimates were reported with 95% uncertainty intervals (UIs) generated from 1000 Monte Carlo simulations. Age-standardized indicators, Joinpoint regression, inequality analysis, decomposition analysis, and frontier analysis were used to assess spatiotemporal trends, drivers, and disparities. Seven forecasting models were evaluated to predict trends from 2022 to 2036. Between 1990 and 2021, global OEA SEV increased from 0.408% to 0.496% (average annual percentage change [AAPC] = 0.288). Cancer deaths rose from 5.5 to 10.5 thousand, YLD from 1.5 to 3.3 thousand, and DALYs from 169.6 to 298.7 thousand. In contrast, age-standardized death rate (AAPC = −0.300) and DALYs rate (AAPC = −0.531) declined. Men bore higher overall burdens, but women experienced faster increases (AAPC 0.428 vs 0.171). High-socio-demographic index (SDI) countries showed continuous improvement, whereas middle- and low-SDI regions-particularly East, South, and Southeast Asia-experienced substantial increases. In East Asia, the AAPCs for age-standardized SEVs, age-standardized death rates, age-standardized YLDs rates, and DALYs rates were 0.953, 1.067, 1.849, and 0.809, respectively. Inequality analysis indicated stable absolute gaps but reduced relative disparities, with burdens still concentrated in low-SDI countries. Decomposition suggested that population growth was the main driver of global burden increases, and frontier analysis highlighted limited improvement potential in low-SDI regions. Forecasting models indicated that the absolute burden of occupational arsenic exposure will continue to rise over the next 15 years. The cancer burden attributable to OEA is still increasing globally, with persistent prevention gaps and socioeconomic disparities. Focused interventions in low- and middle-SDI countries and rapidly industrializing regions are needed, including strengthened occupational exposure regulations, improved workplace arsenic monitoring, engineering controls, personal protective equipment, and regular health surveillance for workers in high-risk industries, to reduce inequalities and mitigate public health threats.

## 1. Introduction

Arsenic is a toxic metalloid, and inorganic arsenic and its compounds are recognized as Group 1 human carcinogens by the International Agency for Research on Cancer.^[1,2]^ Occupational exposure to arsenic (OEA) primarily occurs via inhalation in workplace settings and is common in industries such as glass manufacturing, metal ore mining, nonferrous metal production and processing (excluding aluminum), steel production, and agriculture.^[3]^ Compared with general environmental exposure through drinking water or irrigation, occupational arsenic exposure is typically of higher intensity, longer duration, and predominantly affects working-age populations, thereby not only increasing individual disease risk but also posing potential threats to societal productivity and economic development.^[4]^ Epidemiological and experimental studies have demonstrated significant associations between OEA and malignancies such as lung cancer, skin cancer, and bladder cancer.^[5]^ Evidence from occupational cohort studies and quantitative reviews indicates that a cumulative exposure-response relationship exists between occupational arsenic exposure and the risk of lung cancer and other cancers.^[6,7]^ Long-term follow-up studies also indicate that arsenic-related cancers may have prolonged latency, with excess risks persisting for decades after exposure reduction.^[8]^ In addition, arsenic exposure has been linked to cardiovascular diseases, diabetes, and respiratory disorders, contributing to substantial public health burdens.^[9]^

Globally, arsenic exposure has been reported to cause considerable disease burden. A study in Bangladesh in 2004 estimated that health loss attributable to drinking water arsenic exposure amounted to 174,174 disability-adjusted life years (DALYs).^[10]^ Similarly, a study in Iran in 2025 reported approximately 3347 cancer cases associated with dietary arsenic exposure, corresponding to 72,606 DALYs.^[11]^ Meanwhile, the Global Burden of Disease (GBD) studies have quantified the impact of occupational carcinogen exposures, including OEA. The GBD 2016 results indicated that approximately 348,741 cancer deaths were attributable to occupational exposure to 14 carcinogens.^[12]^ The GBD 2017 study further reported that all occupational carcinogens collectively caused 319,000 cancer deaths and 6.42 million DALYs, with asbestos, silica, and diesel engine exhaust contributing most significantly.^[13]^ However, these GBD-based assessments mainly examined occupational carcinogens collectively or emphasized major contributors such as asbestos, silica, and diesel engine exhaust. OEA-specific analyses of long-term global trends, regional and national disparities, socioeconomic inequalities, and future burden projections remain scarce.

Despite increasing recognition of arsenic-related health hazards, most existing studies have focused on specific regions or primarily on environmental exposure via drinking water and diet, with long-term trends and global comparisons of disease burden attributable to OEA remaining limited. Considerable variations may exist among countries, regions, and sexes in occupational arsenic exposure levels and the cancer burden attributable to it, while predictive studies on future disease burden are relatively scarce. Furthermore, compared with traditional time-series models such as ARIMA, machine learning approaches can better accommodate nonlinear patterns, complex temporal dynamics, and potential interactions in disease burden data, thereby providing a flexible framework for forward-looking public health assessments.^[14]^ However, their application in OEA-related burden prediction remains limited. To address these gaps, this study used GBD 2021 data from 1990 to 2021 to systematically quantify global, regional, and national trends in OEA exposure and attributable cancer burden, thereby filling the gap in long-term OEA-specific assessments. We further examined differences by age, sex, region, and socio-demographic index (SDI) level, and applied inequality, decomposition, and frontier analyses to clarify socioeconomic disparities, key drivers, and potential improvement gaps. Finally, we compared multiple machine learning models to project future trends through 2036, addressing the scarcity of forward-looking evidence for OEA-related cancer burden and providing scientific support for targeted occupational health protection and arsenic exposure mitigation policies.

## 2. Methods

### 2.1. Data sources

Data for this study were obtained from the Global Burden of Disease Study 2021 (GBD 2021), specifically the risk estimates related to OEA.^[15]^ OEA primarily refers to long-term exposure of workers to inorganic arsenic and its compounds in occupational settings such as mining, smelting, wood preservation, glass manufacturing, and the semiconductor industry. The GBD database, developed and maintained by the Institute for Health Metrics and Evaluation at the University of Washington, is a leading source of disease burden data and is publicly accessible through the Global Health Data Exchange. The GBD 2021 database includes data from 204 countries and territories, covering 371 diseases and injuries and 88 risk factors, with estimates stratified across 21 geographic regions and 5 SDI levels.

Data were downloaded from the GBD Results Tool using the GBD 2021 data version. The GBD Results Tool was queried using the following filters: OEA as the risk factor; SEVs, deaths, years lived with disability (YLDs), and DALYs as measures; number, rate, and percent as metrics where applicable; 1990 to 2021 as the study years; global, 21 GBD regions, 204 countries and territories, and 5 SDI categories as locations; both sexes, male, and female as sex categories; and all ages, age-standardized,and available age-specific groups as age categories. For burden estimates, cancer-related outcomes attributable to OEA were extracted according to the GBD 2021 comparative risk assessment framework. These indicators were used to evaluate spatiotemporal trends and differences in OEA-related exposure and attributable cancer burden across sex, age, region, country, and socioeconomic development level. This study used publicly available, aggregated, and de-identified data from the Global Burden of Disease Study 2021 and did not involve individual-level human participant data. Therefore, ethical approval and informed consent were not required.

### 2.2. Estimation methods

In GBD studies, OEA is classified as an occupational carcinogen and treated as a risk factor, with cancer as the primary health outcome. Exposure assessment follows a comparative risk assessment framework. First, data from the International Labor Organization labor force surveys, national censuses, and official and industry-specific statistics were used to estimate occupational exposure proportions across industries and populations. Second, workplace arsenic concentration measurements and exposure data reported in epidemiological literature were incorporated. For regions lacking empirical measurements, stratified modeling and data extrapolation from neighboring countries or comparable regions were applied to supplement data, allowing derivation of exposure distributions and aggregated SEVs. Relative risks were obtained from systematic reviews and meta-analyses to establish dose-response relationships between OEA and cancer incidence. Population attributable fractions (PAFs) were calculated based on exposure distributions and relative risks. PAF represents the proportion of cancer burden attributable to OEA under the comparative risk assessment framework by comparing the observed exposure distribution with the theoretical minimum risk exposure level. OEA-attributable cancer deaths and DALYs were then estimated by multiplying total cancer deaths and DALYs by the corresponding PAFs. Detailed formulas are provided in Appendix 1, Supplemental Digital Content 1, Formula 1. All results were generated using 1000 Monte Carlo simulations to obtain means and 95% uncertainty intervals (UIs), reflecting uncertainty in estimates. Detailed methodologies are described in previous GBD publications.^[16,17]^

### 2.3. Statistical analysis

The study comprehensively considered exposure magnitude, risk levels, and the severity of risk-related disease burden, using SEVs as the primary metric (range: 0–100%; formula in Appendix 1, Supplemental Digital Content 1, Formula 2). To account for differences in population age structure, direct age-standardization was performed using the GBD world standard population, generating age-standardized summary exposure values (ASSEVs), age-standardized death rates (ASDRs), age-standardized YLD rates (ASYLDs), and age-standardized DALY rates (ASDALYs) (formulas in Appendix 1, Supplemental Digital Content 1, Formula 3). Temporal trends were analyzed using Joinpoint regression software (version 5.1.0.0, National Cancer Institute), estimating annual percentage change and average annual percentage change (AAPC) for each indicator, with 95% UIs and 95% confidence intervals (CIs) reported (formulas in Appendix 1, Supplemental Digital Content 1, Formulas 4–5).

Equity analyses were conducted across SDI categories to compare disease burden and trends among countries and regions with different socioeconomic development levels, thereby evaluating global and regional health inequalities. Decomposition analyses further partitioned changes in disease burden into contributions from population size, age structure, and epidemiological factors, quantifying the impact of each component on overall trends. Frontier analyses were employed to identify gaps between countries’ disease burden and their corresponding SDI levels, assessing the effectiveness of disease control and potential room for improvement.

Finally, a time-series forecasting framework was used to assess the trends of each GBD indicator over the next 15 years. To preserve temporal ordering and avoid information leakage, the annual time-series were first split chronologically into 80% training and 20% testing sets to preliminarily evaluate out-of-sample predictive performance. Expanding-window rolling-origin validation was then used as the main basis for model comparison and selection, with an initial training window of 20 years, a 3-year forecasting horizon, and the forecast origin rolled forward by 1 year at a time. Candidate models included ARIMA, exponential smoothing state space model (ETS), linear regression, multivariate adaptive regression splines (MARS), Prophet, elastic net regression, and random forest. Model performance was compared using the average root mean square error (RMSE), mean absolute error (MAE), and mean absolute percentage error (MAPE) from rolling validation.^[18]^ The final model was selected based on lower prediction errors and reasonable trend behavior, and was then refitted using the full historical series for future projection. Given that all indicators were short annual time series, large-scale hyperparameter searches were avoided to reduce the risk of overfitting and unstable long-term extrapolation. Limited tuning was performed only for Elastic Net, MARS, and random forest, and the tuned models were compared with the default or prespecified models. All analyses were conducted using R Studio (version 4.6.0).

## 3. Results

### 3.1. Global burden attributable to occupational arsenic exposure

As shown in Table 1, the global OEA SEV was 0.496% (95% UI: 0.152%–0.925%) in 2021, increasing from 0.408% (95% UI: 0.104%–0.799%) in 1990. During 1990–2021, the ASSEV rose from 0.439% (95% UI: 0.111%–0.861%) to 0.479% (95% UI: 0.148%–0.892%), with an AAPC of 0.288% (95% CI: 0.266%–0.311%). Supplementary Tables S1–S3, Supplemental Digital Content 2 present the changes in deaths, YLDs, and DALYs attributable to OEA over the same period. Globally, the number of deaths increased from 5.5 thousand (95% UI: 0.24–10.41) in 1990 to 10.53 thousand (95% UI: 2.02–18.51) in 2021, whereas ASDRs declined (AAPC = -0.300, 95% CI: −0.554 to −0.046). YLDs increased approximately 1.2-fold, from 1.48 thousand (95% UI: 0.04–3.12) in 1990 to 3.25 thousand (95% UI: 0.58–6.03) in 2021, with a modest rise in ASYLDs (AAPC = 0.142, 95% CI: 0.051–0.233). Global DALYs increased from 169.58 thousand (95% UI: 9.03–318.71) to 298.66 thousand (95% UI: 62.60–523.21), whereas ASDALYs decreased from 3.972 per 100,000 (95% UI: 0.202–7.475) in 1990 to 3.360 per 100,000 (95% UI: 0.704–5.888) in 2021 (AAPC = -0.531, 95% CI: −0.671 to −0.392). Notably, the 95% UIs for global SEVs widened slightly from 1990 to 2021, while the uncertainty ranges for absolute deaths, YLDs, and DALYs also became broader over time, suggesting increased uncertainty in these estimates.

**Table 1:** Age-standardized summary exposure values (SEVs) and the average annual percentage change (AAPC) of occupational exposure to arsenic at the global and regional levels, 1990–2021.

Category	%, Exposure values (95% UI)				AAPC (95% CI)
	Value in 1990	Age-standardized SEVs in 1990	Value in 2021	Age-standardized SEVs in 2021	
**Global**	0.408 (0.104–0.799)	0.439 (0.111–0.861)	0.496 (0.152–0.925)	0.479 (0.148–0.892)	0.288 (0.266–0.311)
**Sex:**					
Male	0.444 (0.117–0.867)	0.473 (0.123–0.926)	0.515 (0.159–0.958)	0.498 (0.154–0.926)	0.171 (0.138–0.203)
Female	0.372 (0.092–0.731)	0.404 (0.099–0.794)	0.478 (0.145–0.893)	0.460 (0.140–0.860)	0.428 (0.389–0.468)
**SDI Level:**					
High	0.526 (0.014–1.239)	0.512 (0.014–1.204)	0.544 (0.028–1.268)	0.491 (0.027–1.144)	−0.136 (−0.168–−0.104)
High-middle	0.448 (0.101–0.907)	0.458 (0.102–0.928)	0.614 (0.179–1.160)	0.540 (0.158–1.019)	0.531 (0.488–0.573)
Middle	0.408 (0.156–0.706)	0.459 (0.175–0.794)	0.568 (0.218–0.983)	0.527 (0.203–0.913)	0.445 (0.406–0.485)
Low-middle	0.295 (0.114–0.508)	0.330 (0.127–0.568)	0.367 (0.139–0.629)	0.381 (0.145–0.654)	0.467 (0.412–0.522)
Low	0.278 (0.108–0.483)	0.317 (0.123–0.550)	0.298 (0.114–0.511)	0.343 (0.132–0.589)	0.256 (0.224–0.288)
**GBD Region:**					
High-income Asia Pacific	0.645 (0–1.581)	0.615 (0–1.507)	0.654 (0–1.591)	0.581 (0–1.412)	−0.188 (−0.223–−0.153)
Western Europe	0.482 (0–1.167)	0.468 (0–1.131)	0.511 (0–1.242)	0.462 (0–1.123)	−0.041 (−0.078–−0.003)
Central Asia	0.307 (0.119–0.525)	0.334 (0.129–0.571)	0.418 (0.159–0.722)	0.393 (0.150–0.680)	0.523 (0.433–0.612)
Southern Latin America	0.422 (0.162–0.729)	0.432 (0.166–0.747)	0.475 (0.183–0.830)	0.459 (0.177–0.802)	0.182 (0.110–0.255)
Australasia	0.440 (0–1.054)	0.435 (0–1.043)	0.478 (0–1.134)	0.439 (0–1.041)	0.033 (−0.025–0.091)
High-income North America	0.516 (0–1.229)	0.507 (0–1.209)	0.502 (0–1.225)	0.465 (0–1.136)	−0.279 (−0.309–−0.248)
Tropical Latin America	0.399 (0.154–0.685)	0.440 (0.170–0.755)	0.457 (0.176–0.803)	0.431 (0.166–0.758)	−0.072 (-0.135–−0.008)
Caribbean	0.333 (0.116–0.591)	0.372 (0.129–0.662)	0.459 (0.165–0.807)	0.447 (0.161–0.787)	0.596 (0.553–0.639)
Central Latin America	0.400 (0.155–0.700)	0.472 (0.184–0.827)	0.523 (0.201–0.899)	0.517 (0.199–0.889)	0.292 (0.269–0.314)
Oceania	0.205 (0.077–0.351)	0.231 (0.087–0.396)	0.261 (0.098–0.454)	0.272 (0.103–0.475)	0.524 (0.481–0.568)
Central Europe	0.492 (0–1.182)	0.468 (0–1.124)	0.545 (0–1.311)	0.482 (0–1.160)	0.098 (0.037–0.158)
Andean Latin America	0.382 (0.145–0.663)	0.449 (0.170–0.777)	0.555 (0.209–0.955)	0.564 (0.213–0.970)	0.737 (0.683–0.790)
Eastern Europe	0.458 (0–1.082)	0.426 (0–1.005)	0.444 (0–1.051)	0.395 (0–0.933)	−0.254 (−0.315–−0.192)
Southeast Asia	0.385 (0.147–0.678)	0.430 (0.163–0.756)	0.561 (0.214–0.957)	0.533 (0.203–0.909)	0.696 (0.664–0.728)
South Asia	0.294 (0.115–0.508)	0.322 (0.126–0.556)	0.332 (0.125–0.576)	0.342 (0.129–0.593)	0.192 (0.163–0.221)
East Asia	0.468 (0.178–0.813)	0.511 (0.194–0.888)	0.803 (0.304–1.376)	0.684 (0.259–1.172)	0.953 (0.929–0.976)
North Africa and Middle East	0.249 (0.097–0.430)	0.287 (0.111–0.496)	0.310 (0.121–0.536)	0.303 (0.118–0.524)	0.182 (0.131–0.232)
Southern Sub-Saharan Africa	0.284 (0.108–0.490)	0.331 (0.126–0.570)	0.200 (0.076–0.343)	0.194 (0.074–0.333)	−1.712 (−1.775–−1.650)
Eastern Sub-Saharan Africa	0.314 (0.120–0.539)	0.372 (0.142–0.640)	0.371 (0.143–0.636)	0.442 (0.170–0.758)	0.558 (0.515–0.601)
Western Sub-Saharan Africa	0.307 (0.118–0.530)	0.352 (0.135–0.607)	0.290 (0.111–0.502)	0.334 (0.128–0.578)	−0.181 (−0.267–−0.096)
Central Sub-Saharan Africa	0.300 (0.114–0.520)	0.348 (0.133–0.603)	0.283 (0.108–0.484)	0.318 (0.122–0.543)	−0.284 (−0.349–−0.219)

AAPC = average annual percentage change, CI = confidence interval, SDI = socio-demographic index, UI = uncertainty interval.

### 3.2. Burden by age and sex

Both male and female ASSEVs showed upward trends globally. In 2021, males had higher ASSEVs than females (0.498% (95% UI: 0.154%–0.926%) vs 0.460% (95% UI: 0.140%–0.860%)). The sex gap narrowed over 30 years, mainly due to the faster increase among females (AAPC: 0.428 vs 0.171). Age- and sex-structured results showed that exposure peaked in the 50 to 54 age group, with females reaching approximately 0.9% (Figure S1A, Supplemental Digital Content 3), suggesting a prominent exposure burden among middle-aged women. Figure S1, Supplemental Digital Content 3, demonstrates pronounced age- and sex-structured differences in SEVs, deaths, YLDs, and DALYs, while Supplementary Tables S1–S3, Supplemental Digital Content 2, provide detailed sex-specific and regional estimates for deaths, YLDs, and DALYs. Deaths were highest in the 70 to 74 age group (Figure S1B, Supplemental Digital Content 3), with male ASDRs decreasing (AAPC = -0.742, 95% CI: −0.915 to −0.568) and female ASDRs increasing (AAPC = 0.744, 95% CI: 0.656–0.831). YLDs peaked in the 65 to 69 age group (Figure S1C, Supplemental Digital Content 3), with male ASYLDs declining from 0.054 per 100,000 (95% UI: 0.001–0.115) in 1990 to 0.049 (95% UI: 0.008–0.091) in 2021, whereas female ASYLDs increased from 0.017 (95% UI: 0.001–0.035) to 0.025 (95% UI: 0.005–0.050) per 100,000. DALYs were also concentrated in the 65 to 69 age group (Figure S1D, Supplemental Digital Content 3), with male ASDALYs decreasing by 26.0% and female ASDALYs increasing by 18.1% over 30 years (AAPC = −0.959 vs 0.530). Despite faster increases among females, males maintained higher levels across all 4 indicators. In addition, the age- and sex-stratified findings further suggested that OEA-attributable cancer burden was mainly concentrated among older adults, particularly older males for mortality- and disability-related indicators, whereas females showed faster temporal increases despite lower overall levels.

### 3.3. Burden by SDI

From 1990 to 2021, all SDI regions except high-SDI areas showed increasing trends (Figure S2, Supplemental Digital Content 4). In 1990, high-SDI regions had the highest ASSEVs, deaths, YLDs, and DALYs globally, but all 4 indicators declined over time (AAPC = -0.136, −1.436, −0.321, −1.748). This decline may partly reflect the earlier implementation of occupational health regulations, workplace exposure monitoring, industrial upgrading, and more effective exposure-control measures in high-SDI settings. By 2021, high-middle SDI regions ranked highest for all indicators, with ASSEVs and YLDs rising (AAPC = 0.531, 0.642), whereas deaths and DALYs decreased (AAPC = −0.007, −0.294). In contrast, the increasing burden in middle- and low-middle-SDI regions may be related to ongoing industrialization, expansion of mining and smelting activities, and relatively limited occupational protection and regulatory enforcement. Low-SDI regions maintained the lowest levels and smallest increases (AAPC = 0.256, 0.200, 0.238, 0.153). Middle-low SDI regions exhibited the largest increases in ASDRs and ASDALYs (AAPC = 0.978 and 0.860), while middle-SDI regions had the highest rise in ASYLDs (AAPC = 1.177) (Table 1; Supplementary Tables S1–S3, Supplemental Digital Content 2).

### 3.4. Burden by region

Between 1990 and 2021, OEA burden varied substantially across 21 GBD regions (Figure S3, Supplemental Digital Content 5). Except for 8 high-income regions, including North America, Central Europe, and high-income Asia Pacific, most regions showed increasing ASSEVs. ASDRs and ASDALYs generally decreased, except in East and South Asia. In 1990, high-income North America had the highest ASDRs, ASYLDs, and ASDALYs, and third-highest SEVs; all 4 indicators declined significantly over 1990–2021 (AAPC = −0.279, −2.399, -1.954, −2.704). By 2021, East Asia ranked highest across all 4 indicators, with continuous increases (AAPC = 0.953, 1.067, 1.849, 0.809). Sub-Saharan Africa remained among the lowest for most indicators, with decreasing ASDRs but slight increases in SEVs, ASYLDs, and ASDALYs (AAPC = −0.181, 0.300, 0.242, 0.182). Over the past 30 years, East Asia consistently exhibited the highest absolute levels of SEVs, deaths, YLDs, and DALYs globally.

### 3.5. Burden by country

**Figure 1.**
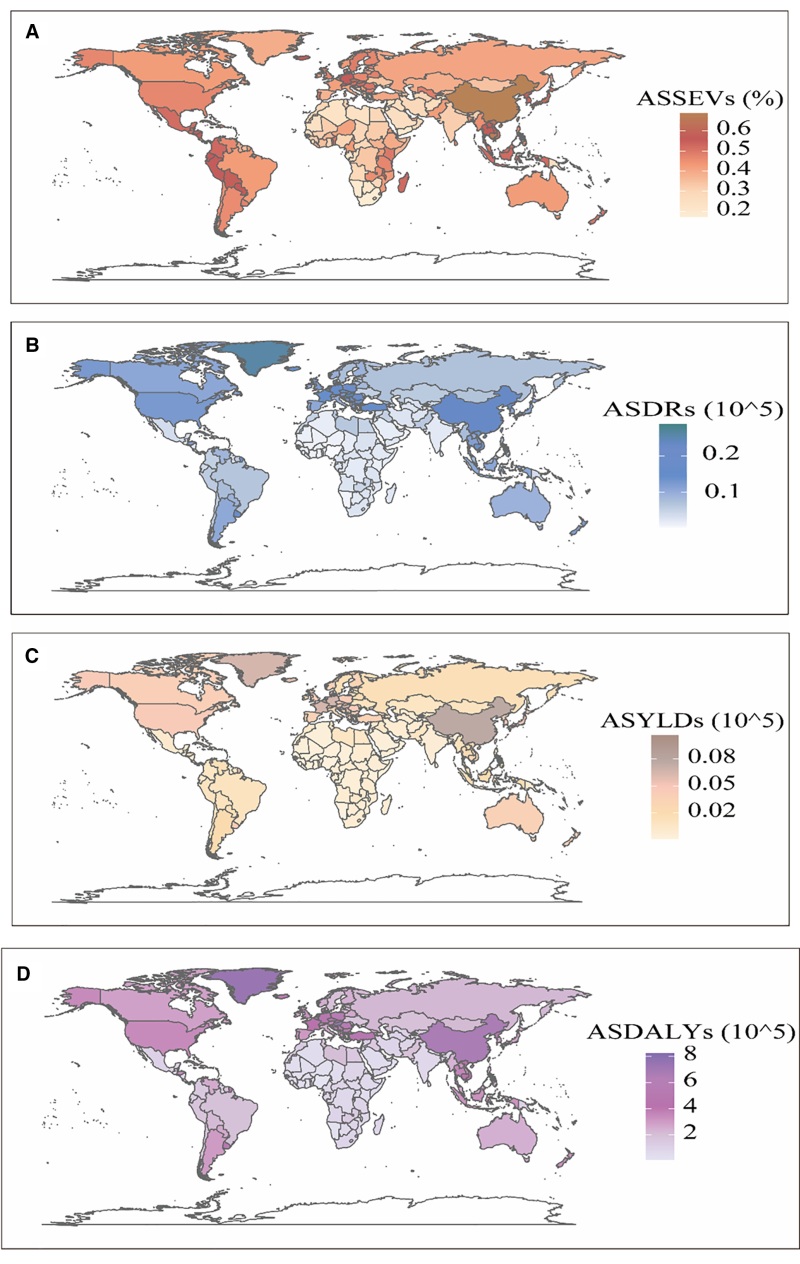
The global burden of OEA in 204 countries in 2021. **(A**) Global distribution of ASSEVs. **(B**) Global distribution of ASDRs. **(C**) Global distribution of ASYLDs. **(D**) Global distribution of ASDALYs. ASDALYs = age-standardized disability adjusted life year rates, ASDRs = age-standardized death rates, ASSEVs = age-standardized summary exposure value, ASYLDs = age-standardized years lived with disability rates, OEA = occupational arsenic exposure.

In 2021, countries with the highest SEVs included China, Vietnam, Cambodia, and the Czech Republic (>0.7%), with China reaching 0.8%. The highest deaths occurred in China (5251), the USA (738), Japan (350), and India (313); highest YLDs were observed in China (1621), the USA (263), Japan (156), and Germany (101); and highest DALYs in China (150,008), the USA (19,131), India (10,112), and Indonesia (9671). China had the highest ASSEV (0.69%), whereas Thailand showed the largest 30-year increase (0.46%) (Fig. 1A). China exhibited the largest rise in ASDRs (0.06 per 100,000), whereas the USA declined significantly, second only to Greenland (−0.14 per 100,000) (Fig. 1B). Morocco had the highest ASYLDs and ASDALYs, with Algeria and Greenland showing the largest decreases (Figs. 1C–D). In absolute numbers, China remained the highest across all 4 indicators, while the USA ranked second for all except SEVs.

### 3.6. Inequality analysis

**Figure 2.**
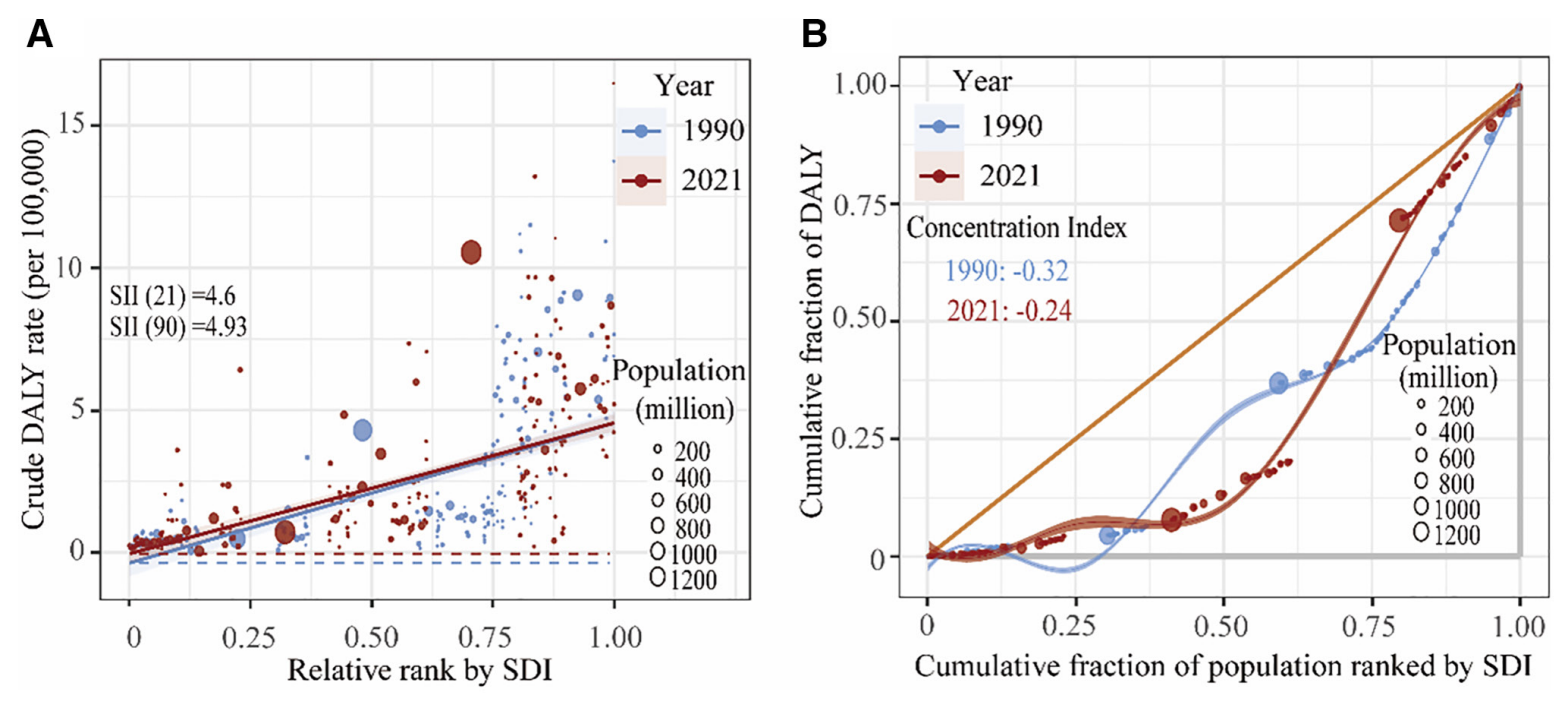
Health inequality analysis of the global burden of OEA. **(A**) Absolute income-related health inequality in the burden of OEA in 1990 compared with 2021. **(B**) The trendline demonstrates the trend in the concentration index from 1990 to 2021. CI = confidence interval, A higher CI value signifies greater inequality, implying that the burden is more concentrated among wealthier populations, OEA = occupational arsenic exposure, SII = slope index of inequality, A higher SII value indicates greater health inequality, reflecting a disproportionately higher health burden among populations with lower socioeconomic status.

Figures 2A and 2B show absolute (slope index of inequality [SII]) and relative (CI) inequalities in ASDALYs stratified by SDI. SII decreased slightly from 4.93 in 1990 to 4.60 in 2021, indicating relatively stable absolute gaps between low- and high-SDI countries. Due to large populations in low-middle SDI regions, DALY burden concentrated in these areas. CI increased from −0.32 in 1990 to −0.24 in 2021, indicating a slight reduction in relative inequality; however, the persistent negative value suggests that low-SDI countries continue to bear disproportionately high burdens.

### 3.7. Decomposition analysis

Figure S4, Supplemental Digital Content 6 shows the drivers of changes in DALYs attributable to OEA from 1990–2021 globally and by SDI. Globally, DALY increases were mainly driven by population growth, with limited contributions from age structure or epidemiological changes; male DALYs were more influenced by epidemiology, whereas female DALYs were predominantly driven by population growth. In high-SDI regions, DALY reductions were mainly due to epidemiological improvements. High-middle SDI increases were primarily population-driven, with negative epidemiological contributions. In middle-SDI regions, population growth and positive epidemiological changes contributed similarly; middle-low SDI increases were mainly population-driven, with males affected by negative epidemiology; low-SDI regions saw slight decreases driven by population size, with consistent trends across sexes.

### 3.8. Frontier analysis

**Figure 3.**
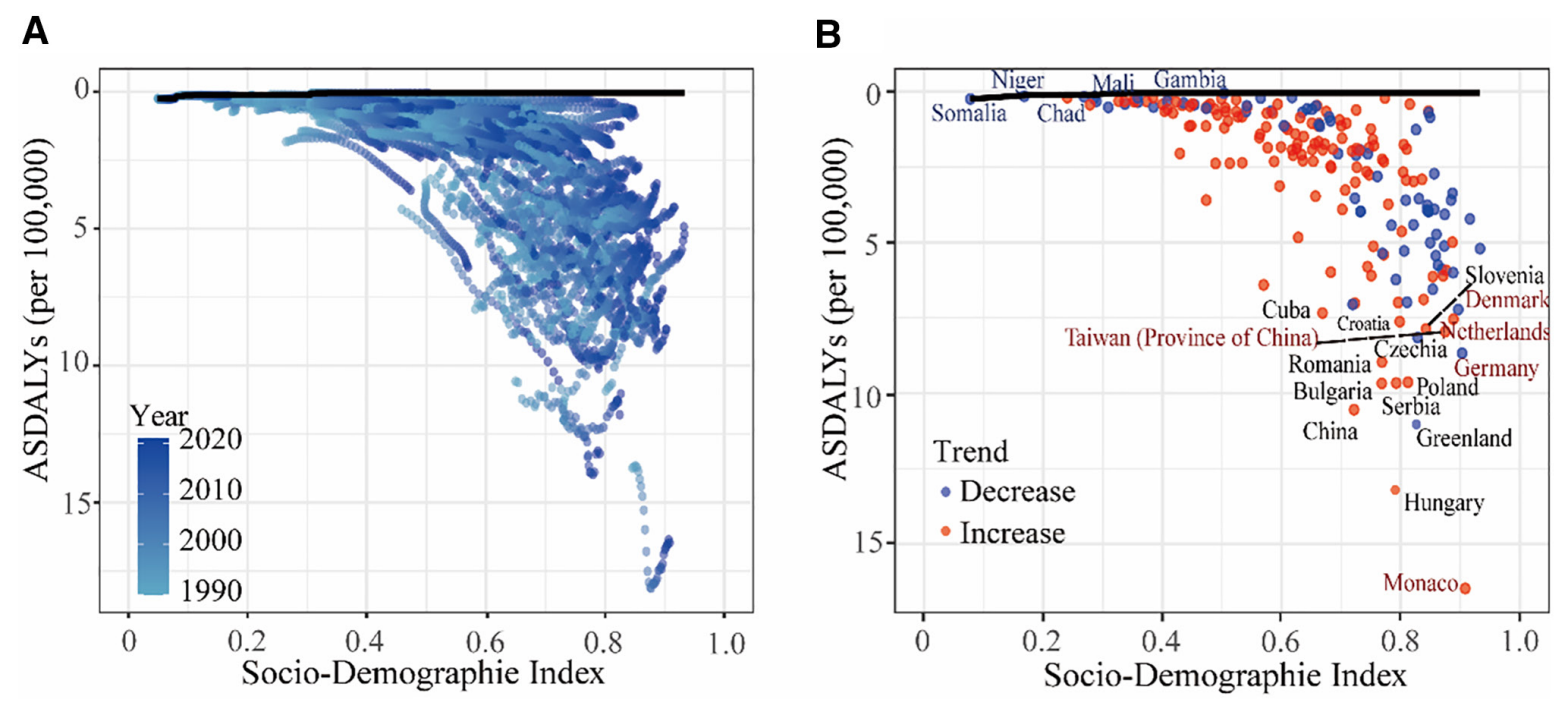
Frontier analysis based on SDI and ASDALYs from 1990 to 2021. **(A**) Frontier analysis based on SDI and ASDALYs from 1990 to 2021. **(B**) Frontier analysis based on SDI and ASDALYs across countries and territories in 2021. ASDALYs = age-standardized disability adjusted life year rates, OEA = occupational arsenic exposure, SDI = socio-demographic index.

From 1990 to 2021, the gap in DALY burden across countries widened with increasing SDI (Fig. 3A). Countries such as China, Poland, and Hungary showed the most prominent effective differences. Figure 3B indicates clear disparities in DALY burden across SDI levels. Effective differences increased with SDI, suggesting greater potential for burden reduction in high-SDI countries. High-SDI countries, including Morocco, Germany, Denmark, and Taiwan, showed higher effective differences, whereas low-SDI countries such as Somalia and Niger exhibited smaller gaps.

### 3.9. Forecast for the next 15 years

**Figure 4.**
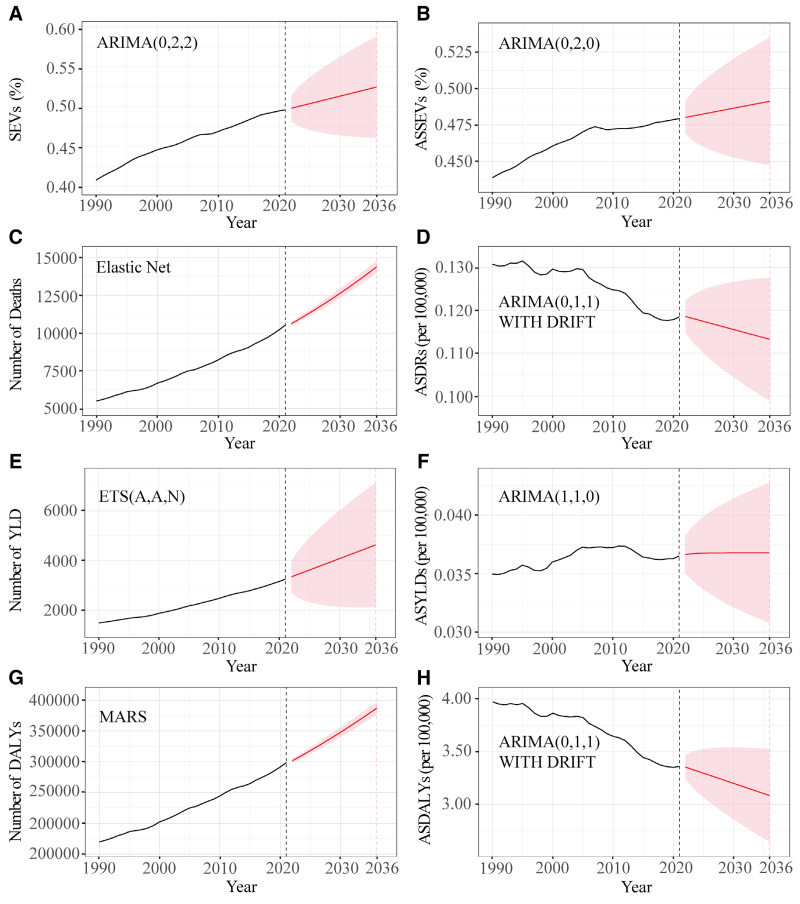
Time-series projections of global OEA disease burden, 2022–2036. **(A**) SEVs, best-fitting model: ARIMA; **(B**) ASSEVs, best-fitting model: ARIMA; **(C**) deaths, best-fitting model: Elastic Net; **(D**) ASDRs, best-fitting model: ARIMA; **(E**) YLD, best-fitting model: ETS; **(F**) ASYLDs, best-fitting model: ARIMA. **(G**) DALYs, best-fitting model: MARS; **(H**) ASDALYs, best-fitting model: ARIMA. ARIMA = autoregressive integrated moving average, ASDALYs = age-standardized disability adjusted life year rates, Elastic Net, elastic net regression, ASDRs = age-standardized death rates, ASSEVs = age-standardized Summary exposure value, ASYLDs = age-standardized years lived with disability rates, DALYs = disability-adjusted life year rates, ETS = exponential smoothing, MARS = multivariate adaptive regression splines, OEA = occupational arsenic exposure, SEVs = summary exposure value, YLD = years lived with disability.

Performance comparisons of 7 models are shown in Tables S4–S5, Supplemental Digital Content 6 and Figure S5, Supplemental Digital Content 7. Lower RMSE, MAE, and MAPE values indicate better predictive accuracy. The optimal models for SEVs, deaths, YLDs, DALYs, ASSEVs, ASDRs, ASYLDs, and ASDALYs were ARIMA, Elastic Net, ETS, MARS, ARIMA, ARIMA, ARIMA, and ARIMA, respectively. For absolute indicators, ARIMA showed the best performance for SEVs, with the lowest RMSE and MAPE (RMSE = 0.0011; MAPE = 0.213), while Elastic Net performed best for deaths (RMSE = 71.035; MAPE = 0.666), ETS for YLDs (RMSE = 22.190; MAPE = 0.704), and MARS for DALYs (RMSE = 1767.213; MAPE = 0.570). For age-standardized indicators, ARIMA consistently showed the best predictive performance for ASSEVs, ASDRs, ASYLDs, and ASDALYs, with RMSE values of 0.0006, 0.0005, 0.0002, and 0.027, and MAPE values of 0.108, 0.313, 0.396, and 0.725, respectively. Forecasts indicate continued increases in absolute indicators (SEVs, deaths, YLDs, DALYs) from 2022 to 2036. For instance, deaths are projected to rise from 10.52 thousand in 2021 to 14.39 thousand (95% CI: 14.0–14.8) in 2036, and DALYs from 298.7 thousand to 387.0 thousand (95% CI: 376.9–397.2). In contrast, relative indicators (ASDRs, ASYLDs, ASDALYs) are expected to decline, e.g., ASDRs from 0.119 per 100,000 in 2021 to 0.113 per 100,000 (95% CI: 0.099–0.128) in 2036 (Fig. 4).

## 4. Discussion

This study systematically evaluated global OEA levels, the cancer burden attributable to it, and temporal trends from 1990 to 2021, revealing substantial spatiotemporal heterogeneity, socioeconomic inequalities, and underlying drivers as well as the effectiveness of control measures. Our findings indicate that despite declines in ASDRs and ASDALYs, overall exposure levels and the absolute disease burden attributable to OEA continue to rise. The burden is unevenly distributed across sex, age, regions, and SDI levels, with significant differences in drivers and the effectiveness of interventions across populations and areas. Machine learning-based forecasts suggest that the absolute burden attributable to OEA will continue to increase, particularly in middle-low and low SDI regions, further highlighting inadequate global OEA control and regional disparities.

Consistent with previous studies,^[13]^ we observed an overall increasing trend in OEA risk over the past 3 decades. The global SEV reached 0.496% in 2021, with an AAPC of 0.288, indicating a steady rise. This trend may be closely linked to global economic growth and accelerated industrialization. Global sectoral employment statistics from ILO/ILOSTAT indicate that industrial employment is concentrated in sectors such as mining and quarrying, manufacturing, construction, and public utilities, which may include occupational settings where arsenic exposure occurs.^[19,20]^ Given the widespread use of arsenic and its compounds in traditional industries, agriculture, and emerging sectors such as semiconductors, potential health risks among working populations may remain substantial, especially where workplace monitoring and exposure-control measures are insufficient.^[4,5,21]^ In addition, occupational hazards and adverse working conditions may also affect workers’ psychological well-being.^[22]^ Concurrently, absolute numbers of deaths and DALYs attributable to OEA have risen, whereas age-standardizedrates declined (AAPC = −0.300 for ASDRs and − 0.531 for ASDALYs). This “increasing absolute burden but declining relative rates” phenomenon likely reflects the role of demographic factors in shaping disease burden: population growth contributes cumulatively to overall disease burden^[23]^; population aging drives increases in cancer cases and DALYs^[24]^; and progress in occupational protection, diagnosis, and treatment,^[12,25]^ as well as birth-cohort effects,^[26]^ has reduced individual-level risk.

Sex-specific analysis revealed that males generally had higher OEA exposure and a greater cancer burden attributable to it than females; however, the past 30 years saw faster increases among females, with deaths, YLDs, and DALYs showing a “male decline, female rise” pattern. This may be associated with higher female labor force participation and greater entry of women in middle-low income regions into high-risk sectors such as mining and manufacturing.^[27,28]^ In addition to these occupational factors, sex-related differences in inorganic arsenic metabolism may also influence susceptibility to arsenic-related health risks. Previous studies have suggested that women generally have higher arsenic methylation efficiency than men, and this difference may be related to sex hormones and metabolic processes involved in arsenic biotransformation.^[29,30]^ Age-specific analysis indicated that the cancer burden attributable to OEA is concentrated in middle-aged and older adults, particularly those aged 50 to 74 years. This pattern is consistent with the long latency of arsenic-induced cancers and cumulative exposure effects,^[8,31]^ suggesting that occupational protection should begin early in the working life and include continuous health monitoring and screening programs.

At regional and national levels, high-SDI countries generally showed declining burdens, notably in the USA and Germany, likely due to strict occupational safety regulations, comprehensive environmental monitoring, and industrial restructuring.^[32,33]^ In contrast, middle-high and middle-low SDI regions experienced substantial increases, particularly in East, Southeast, and South Asia. Countries such as China, India, Bangladesh, Vietnam, and Thailand had the highest exposure levels, deaths, and DALYs. This trend may be driven not only by global industrial shifts, rapid industrialization, concentrated mining and smelting industries, and insufficient occupational protection,^[3,18]^ but also by widespread naturally high arsenic concentrations in groundwater.^[34]^ Previous studies have reported elevated inorganic arsenic in groundwater in regions such as the Ganges-Brahmaputra-Meghna basin, Red River Delta in Vietnam, and Mekong River plains in Cambodia,^[35,36]^ potentially resulting in combined occupational and environmental exposure and increasing chronic health risks, including cancer. However, although Sub-Saharan African regions showed relatively low estimated OEA-attributable cancer burdens in our analysis, this finding should be interpreted with caution. Localized arsenic exposure risks may still exist in parts of Africa, particularly in mining-related settings such as artisanal and small-scale gold mining, mining-related dust, tailings, and contaminated water. These local risks may not be fully captured by regional average estimates, especially where occupational exposure monitoring and disease surveillance remain limited.^[37,38]^

Our findings also show that trends in the cancer burden attributable to OEA are jointly driven by population growth and epidemiological changes, with marked socioeconomic inequalities. Over the past 30 years, absolute disparities between high- and low-income countries remained relatively stable (SII: 4.93 to 4.60), while relative inequalities improved slightly (CI: −0.32 to −0.24), yet the burden remains concentrated in low-SDI countries.^[39]^ Decomposition analysis indicated that global DALY increases were mainly population-driven, whereas epidemiological improvements predominantly benefited high-SDI regions. In middle-low SDI regions, growth was almost entirely driven by population, with some negative epidemiological effects among males, highlighting that in resource-limited settings, demographic dividends alone cannot improve health outcomes.^[40]^ Frontier analysis further revealed that disparities across countries widen with increasing SDI, with high-SDI countries having greater potential for improvement, whereas low-SDI countries face limited scope, emphasizing the need for international cooperation and resource support.^[41]^ Notably, countries such as China, Poland, and Hungary exhibited significant gaps, suggesting opportunities for substantial improvement. These inequality findings have important policy implications and are closely aligned with global occupational health and sustainable development frameworks. In particular, SDG 8.8 emphasizes the protection of labor rights and the promotion of safe and secure working environments for all workers, while WHO/ILO occupational health frameworks highlight the importance of workplace health protection, access to occupational health services, evidence-based action, and cross-sectoral policy integration. Therefore, future interventions should focus on low-SDI and high-burden regions by strengthening occupational exposure monitoring, regulatory enforcement, occupational health services, environmental management, and industrial restructuring to reduce global health inequalities.^[42,43]^

Forecasting analysis suggested that over the next 15 years, OEA levels and absolute cancer deaths and DALYs will continue to rise, whereas age-standardized rates will decline. The persistence of this “rising absolute burden but declining relative rate” trend indicates that population aging will continue to drive disease burden increases, while overall population growth further amplifies total burden. Meanwhile, improvements in individual-level occupational health protection, early diagnosis, and medical services may reduce personal disease risk, contributing to declines in relative rates. However, without systematic and stringent occupational health policy interventions, particularly in middle-low and low SDI countries and rapidly industrializing regions, the cancer burden attributable to OEA is expected to continue rising. These forecasts should therefore be interpreted as reference projections based on the continuation of historical temporal patterns, rather than estimates under specific intervention scenarios. If occupational exposure control, workplace monitoring, regulatory enforcement, and industrial upgrading are strengthened, future OEA-attributable cancer burden may deviate downward from the projected trends. Conversely, delayed implementation of preventive measures or continued expansion of high-risk industries could result in higher-than-expected burdens. Importantly, to enhance the reliability of predictions and fully capture the complexity of disease burden evolution, this study employed multiple machine learning models, selecting the optimal model through cross-validation and multi-metricevaluation. Unlike prior studies relying mainly on single time-series models (e.g., ARIMA or BAPC) ^[44,45]^, we compared 7 common algorithms, including ARIMA, ETS, MARS, Prophet, Elastic Net, and Random Forest, allowing linear and nonlinear features to be captured within a unified framework and adequately representing temporal trends and multifactor interactions.^[46]^ Machine learning models offer advantages in feature extraction and nonlinear fitting, achieving higher predictive accuracy in complex 11, multidimensional OEA and disease burden data. The results revealed that optimal models differed across indicators, reflecting the diverse and complex temporal evolution of disease burden.

## 5. Limitations

This study has several limitations. First, the data were derived from the GBD study, which, although extensive and internationally comparable, relies on modeling assumptions and secondary data sources, introducing potential uncertai0nty. In particular, estimates of occupational arsenic exposure may be affected by incomplete workplace monitoring, sparse exposure measurements, and underreporting of occupational diseases, especially in low-SDI regions and informal or small-scale sectors where routine surveillance is limited. Second, this study focused on cancer burden attributable to occupational arsenic exposure and did not quantify non-cancer outcomes. Within the extracted GBD 2021 risk-outcome framework, the attributable burden of occupational arsenic exposure was limited to cancer outcomes, and non-cancer outcomes attributable specifically to occupational arsenic exposure were not directly available. Although previous burden-of-disease studies of nonoccupational inorganic arsenic exposure have suggested that non-cancer outcomes, particularly cardiovascular and metabolic diseases, may account for a substantial proportion of arsenic-related DALYs,^[47]^ these proportions cannot be directly applied to occupational arsenic exposure because of differences in exposure pathways, populations, and risk-outcome definitions. Therefore, our cancer-focused analysis may underestimate the broader health burden of occupational arsenic exposure, and future studies should further quantify both cancer and non-cancer outcomes attributable specifically to occupational arsenic exposure. Third, forecasts were mainly based on historical temporal patterns and did not explicitly incorporate external drivers such as future occupational health policies, technological progress, regulatory enforcement, industrial restructuring, or changes in workplace exposure-control measures. Therefore, the projected trends should be interpreted cautiously as reference estimates rather than intervention-specific forecasts. Future studies could improve prediction frameworks by developing hybrid or scenario-based models that integrate time-series methods, machine learning algorithms, and policy- or technology-related covariates, thereby better capturing the potential effects of interventions and structural changes on future OEA-attributable cancer burden. Finally, our projections did not include scenario-based sensitivity analyses. Because intervention coverage, compliance, exposure reduction magnitude, industrial transition pathways, and the latency between exposure reduction and cancer outcomes vary substantially across countries, these global- or country-level data are difficult to obtain and are not directly available from GBD 2021. Therefore, we did not construct quantitative intervention scenarios. The projected trends should be interpreted as historical-trend reference estimates rather than intervention-specific forecasts. Future studies should incorporate policy and intervention scenarios to better quantify the potential impact of occupational arsenic exposure reduction strategies.

## 6. Conclusions

This study provides a comprehensive estimation of the global cancer burden attributable to OEA. From 1990 to 2021, global SEVs and absolute cancer deaths and DALYs generally increased, whereas age-standardized rates declined. These divergent trends suggest that population growth and aging continue to drive the absolute burden despite improvements in relative rates. The burden remains unevenly distributed across sex, regions, and SDI levels, with persistent socioeconomic inequalities. Forecasts indicate that, without effective interventions, the burden may continue rising in low-middle and low-SDI countries and rapidly industrializing regions. Priority should be given to immediate workplace exposure control and occupational health protection in high-burden and rapidly industrializing regions, particularly parts of Asia. In line with international occupational health frameworks, such as ILO conventions on occupational safety and chemical safety at work and the WHO Global Plan of Action on Workers’ Health, strengthened workplace monitoring, regulatory enforcement, technical support, and global collaboration are urgently needed to mitigate the public health threat posed by occupational arsenic exposure.

## Author contributions

**Data curation:** Di Wu, Xinxiao Li, Minghan Luo, Nian Gao, Xiaowei Wang, Ailin Li, Zhe Mo, Zafar Gazala, Shirui Yan, Lei Wu, Rui Zhang, Junrui Pei.

**Formal analysis:** Di Wu, Xinxiao Li, Minghan Luo, Nian Gao, Xiaowei Wang, Ailin Li, Zhe Mo, Zafar Gazala, Shirui Yan, Lei Wu, Rui Zhang, Junrui Pei.

**Methodology:** Di Wu, Junrui Pei.

**Resources:** Di Wu.

**Software:** Di Wu, Xinxiao Li.

**Supervision:** Di Wu, Junrui Pei.

**Visualization:** Di Wu, Xinxiao Li, Junrui Pei.

**Conceptualization:** Junrui Pei.

**Writing – original draft:** Di Wu, Xinxiao Li, Junrui Pei.

**Writing – review & editing:** Di Wu, Xinxiao Li, Junrui Pei.














