# Supplementary material for: Cancer burden and health inequalities attributable to occupational arsenic exposure: A 32-year global, regional, and national observational study with projections to 2036
Source: Medicine (Baltimore). 2026 Jul 31;105(31):e49979. doi: 10.1097/MD.0000000000049979 (PMC13433024; doi:10.1097/MD.0000000000049979)
Supplement: Supplementary file 1 [file medi-105-e49979-s001.docx]

**Appendix 1: Calculation Formulas**

**Formula 1: Population Attributable Fraction (PAF)** ^1^:

$$\begin{aligned} PAF=\frac{\sum_{x=1}^{n} P\left( X \right)RR\left( X \right)-1}{\sum_{x=1}^{n} RR\left( X \right)P\left( X \right)} \end{aligned}$$

Where $P\left( X \right)$ represents the exposure distribution and $RR\left( X \right)$ denotes the relative risk associated with each exposure level. This formula estimates the proportion of cases or deaths from a specific cancer that can be attributed to arsenic exposure.

**Formula 2: Summary Exposure Values (SEVs)** ^2^:

$$\begin{aligned} SEVs=\frac{\sum_{i=1}^{n} P_{i}\left( {RR}_{i}-1 \right)}{{RR}_{max}-1}\times100\% \end{aligned}$$

Where $P_{i}$ is the proportion of the population at risk level $i$ , ${RR}_{i}$ is the relative risk at that level, ${RR}_{max}$ ​is the maximum relative risk across all levels, and $n$ is the number of risk levels. SEVs range from 0 (no excess risk in the population) to 1 (entire population at maximum risk) and are reported as percentages to reflect risk-weighted exposure.

**Formula 3: Age-Standardized Rates (ASR)** ^3^:

$$\begin{aligned} ASR=\frac{\sum_{i=1}^{n} \left( a_{i}\times w_{i} \right)}{\sum_{i=1}^{n} \left( w_{i} \right)} \end{aligned}$$

Where $a_{i}$​ is the age-specific rate for age group $i$, $w_{i}$​ is the standard population in age group $i$, and $n$ is the number of age groups. ASRs are typically expressed per 100,000 population and are used for ASSEVs, ASDRs, ASYLDs, and ASDALYs.

**Formula 4 & 5: Annual Percentage Change (APC) and Average Annual Percentage Change (AAPC)** ^4^:

$$\begin{aligned} APC=\left( e^{\beta_{1}}-1 \right)\times100 \end{aligned}$$

Where $\beta_{1}$​ is the slope from a log-linear regression model representing the annual change rate. For multiple segments, AAPC is calculated as ^5^:

$$\begin{aligned} AAPC=\left( e^{\frac{\sum w_{i}\beta_{i}}{\sum w_{i}}}-1 \right)\times100 \end{aligned}$$

Where $w_{i}$ is the segment weight and $\beta_{1}$​ is the slope for segment $i$.

**Formula 6: ARIMA (p,d,q) Model** ^6,7^:

$$\begin{aligned} \Delta^{d}x_{t}=\emptyset_{1}\Delta^{d}x_{t-1}+\ldots+\emptyset_{p}\Delta^{d}x_{t-p}+\varepsilon_{t}+\theta_{1}\varepsilon_{t-1}+\ldots+\theta_{q}\varepsilon_{t-q} \end{aligned}$$

Where $\Delta^{d}x_{t}$ is the $d$ order differenced series, $\emptyset_{i}$​ are autoregressive coefficients, $\theta_{j}$ are moving average coefficients, and $\varepsilon_{t}$​ is white noise. ARIMA models capture trends and random fluctuations in stationary or differenced time series.

**Formula 7: ETS (Exponential Smoothing) Model** ^7^:

$$\begin{aligned} \hat{y_{t+1}}=\alpha y_{t}+(1-\alpha)\hat{y_{t}} \end{aligned}$$

Where $\hat{y_{t+1}}$ ​ is the forecast for time $t+1$, $y_{t}$​ is the observed value at time $t$, $\hat{y_{t}}$​ is the forecast at time $t$, and $0＜\alpha＜1$ is the smoothing parameter. ETS assigns exponentially decreasing weights to historical observations, smoothing the series and enabling effective short-term forecasting of stationary time series.

**Formula 8: Linear Regression Model** ^8^:

$$\begin{aligned} Y_{i}=\beta_{0}+\beta_{1}X_{i}+\varepsilon_{i} \end{aligned}$$

Where $Y_{i}$ is the dependent variable for observation $i$, $X_{i}$​ is the independent variable, $\beta_{0}$ is the intercept, $\beta_{1}$ is the regression coefficient, and $\varepsilon_{i}$ is the error term. Linear regression describes the linear relationship between a dependent variable and one or more predictors and is widely used for prediction and variable relationship analysis.

**Formula 9: MARS (Multivariate Adaptive Regression Splines) Model** ^9^:

$$\begin{aligned} \hat{Y}=\beta_{0}+\sum_{m=1}^{M} \beta_{m}B_{m}(X) \end{aligned}$$

Where $\hat{Y}$ is the predicted value, $\beta_{0}$​ is the intercept, $\beta_{m}$​ are regression coefficients, and $B_{m}(X)$ are basis functions (typically products of hinge functions). MARS is a non-parametric regression method that captures complex nonlinear and non-additive relationships in the data.

**Formula 10: Prophet Model** ^7^:

$$\begin{aligned} y\left( t \right)=g\left( t \right)+s\left( t \right)+h\left( t \right)+\varepsilon_{i} \end{aligned}$$

Where $y\left( t \right)$ is the observed value at time $t$, $g\left( t \right)$ is the trend component (linear or logistic growth), $s\left( t \right)$ is the seasonal component (modeled using Fourier series), $h\left( t \right)$ is the holiday effect, and $\varepsilon_{i}$ is the error term. Prophet decomposes the time series into trend, seasonality, and holiday effects for effective forecasting of non-stationary series.

**Formula 11: Elastic Net Regression Model** ^10^:

$$\begin{aligned} \hat{\beta}=\arg\begin{matrix} min \\ \beta\end{matrix}\left\{ \parallel y\boldsymbol{-}X\beta\parallel_{2}^{2}\boldsymbol{+}\lambda_{1}\parallel\beta\parallel_{1}\boldsymbol{+}\lambda_{2}\parallel\beta\parallel_{2}^{2}\boldsymbol{} \right\} \end{aligned}$$

Where $y$ is the response vector, $X$ is the predictor matrix, $\beta$ is the coefficient vector, $\parallel\cdot\parallel_{1}$​ is the L1 norm (Lasso regularization), $\parallel\cdot\parallel_{2}$​ is the squared L2 norm (Ridge regularization), and $\lambda_{1},\lambda_{2}$​ are regularization parameters. Elastic Net combines Lasso and Ridge regularization to handle high-dimensional data and correlated predictors while preventing overfitting.

**Formula 12: Random Forest Regression Model** ^11^:

$$\begin{aligned} \hat{f}_{RF}\left( x \right)=\frac{1}{B}\sum_{b=1}^{B} T_{b}(x) \end{aligned}$$

Where $x$ is the input feature vector, $T_{b}\left( x \right)$ is the prediction from the $b$ tree, and $B$ is the total number of trees in the forest. Random Forest is an ensemble learning method that improves prediction accuracy, reduces overfitting, and evaluates variable importance, suitable for high-dimensional and nonlinear data.

**References**

1. LEVIN ML. The occurrence of lung cancer in man. *Acta Unio Int Contra Cancrum*. 1953;9(3):531-541.
2. GBD 2017 Risk Factor Collaborators. Global, regional, and national comparative risk assessment of 84 behavioural, environmental and occupational, and metabolic risks or clusters of risks for 195 countries and territories, 1990-2017: a systematic analysis for the Global Burden of Disease Study 2017. *Lancet*. 2018;392(10159):1923-1994. doi:10.1016/S0140-6736(18)32225-6
3. GBD 2019 Risk Factors Collaborators. Global burden of 87 risk factors in 204 countries and territories, 1990-2019: a systematic analysis for the Global Burden of Disease Study 2019. *Lancet*. 2020;396(10258):1223-1249. doi:10.1016/S0140-6736(20)30752-2
4. National Cancer Institute. Annual Percent Change (APC) and Confidence Interval. Accessed July 25, 2025. https://surveillance.cancer.gov/help/joinpoint/setting-parameters/method-and-parameters-tab/apc-aapc-tau-confidence-intervals/estimate-average-percent-change-apc-and-confidence-interval
5. National Cancer Institute. Average Annual Percent Change (AAPC) and Confidence Interval. Accessed July 25, 2025. <https://surveillance.cancer.gov/help/joinpoint/setting-parameters/method-and-parameters-tab/apc-aapc-tau-confidence-intervals/average-annual-percent-change-aapc>
6. Box GE, Jenkins GM, Reinsel GC, Ljung GM. *Time Series Analysis: Forecasting and Control*. 5th ed. Hoboken, NJ: Wiley; 2015.
7. Hyndman RJ, Athanasopoulos G. *Forecasting: Principles and Practice*. 3rd ed. Melbourne, Australia: OTexts; 2021.
8. Montgomery DC, Peck EA, Vining GG. Introduction to Linear Regression Analysis. 6th ed. Hoboken, NJ: John Wiley & Sons; 2021.
9. James G, Witten D, Hastie T, Tibshirani R. *An Introduction to Statistical Learning: with Applications in R*. New York, NY: Springer; 2013.
10. Zou H, Hastie T. Regularization and variable selection via the elastic net. *J R Stat Soc Series B Stat Methodol*. 2005;67(2):301-320. doi:10.1111/j.1467-9868.2005.00503.x.

Breiman L. Random forests. *Mach Learn*. 2001;45:5–32.
