# Supplementary material for: Cancer burden and health inequalities attributable to occupational arsenic exposure: A 32-year global, regional, and national observational study with projections to 2036
Source: Medicine (Baltimore). 2026 Jul 31;105(31):e49979. doi: 10.1097/MD.0000000000049979 (PMC13433024; doi:10.1097/MD.0000000000049979)
Supplement: Supplementary file 2 [file medi-105-e49979-s002.doc]

**Table S1.** Age-standardized death rates (ASDRs) and the average annual percentage change (AAPC) of cancer deaths attributable to occupational arsenic exposure at the global and regional levels, 1990-2021.

|  | **Death rates (95% UI)** | | | | **AAPC (95% CI)** |
| --- | --- | --- | --- | --- | --- |
| **Category** | **Cases in 1990 (thousand)** | **ASDRs in 1990  (per 100,000)** | **Cases in 2021 (thousand)** | **ASDRs in 2021 (per 100,000)** |
| **Global** | 5.50 (0.24-10.41) | 0.131 (0.005-0.248) | 10.53 (2.02-18.51) | 0.119 (0.023-0.208) | -0.300 (-0.554 - -0.046) |
| **Sex:** |  |  |  |  |  |
| Male | 4.15 (0.15-7.90) | 0.205 (0.007-0.392) | 6.92 (1.25-12.36) | 0.162 (0.029-0.290) | -0.742 (-0.915 - -0.568) |
| Female | 1.35 (0.10-2.51) | 0.062 (0.004-0.115) | 3.61 (0.78-6.43) | 0.078 (0.017-0.139) | 0.744 (0.656-0.831) |
| **SDI Level:** |  |  |  |  |  |
| High* | 2.179 (0-4.605) | 0.203 (0-0.429) | 2.553 (-0.403-5.281) | 0.130 (0-0.269) | -1.436 (-1.564 - -1.307) |
| High-middle | 1.827 (0.143-3.425) | 0.172 (0.014-0.322) | 3.471 (0.860-6.018) | 0.171 (0.043-0.296) | -0.007 (-0.259-0.246) |
| Middle | 1.223 (0.456-1.980) | 0.105 (0.039-0.171) | 3.735 (1.354-6.267) | 0.130 (0.047-0.217) | 0.688 (0.502-0.874) |
| Low-middle | 0.213 (0.076-0.357) | 0.031 (0.011-0.052) | 0.647 (0.238-1.057) | 0.041 (0.015-0.068) | 0.978 (0.898-1.057) |
| Low | 0.047 (0.017-0.084) | 0.018 (0.006-0.032) | 0.111 (0.040-0.190) | 0.019 (0.007-0.033) | 0.200 (0.039-0.360) |
| **GBD Region:** |  |  |  |  |  |
| High-income Asia Pacific* | 0.305 (0-0.642) | 0.145 (0-0.306) | 0.460 (0-0.995) | 0.109 (0-0.236) | -0.961 (-1.306 - -0.616) |
| Western Europe* | 1.003 (0-2.151) | 0.186 (0-0.399) | 1.054 (0-2.274) | 0.129 (0-0.278) | -1.206 (-1.319 - -1.093) |
| Central Asia | 0.060 (0.023-0.097) | 0.114 (0.043-0.185) | 0.046 (0.017-0.078) | 0.049 (0.018-0.081) | -2.700 (-2.858 - -2.542) |
| Southern Latin America | 0.075 (0.028-0.124) | 0.159 (0.058-0.262) | 0.081 (0.030-0.133) | 0.095 (0.035-0.157) | -1.583 (-1.745 - -1.420) |
| Australasia* | 0.036 (0-0.078) | 0.155 (0-0.333) | 0.050 (0-0.109) | 0.098 (0-0.213) | -1.473 (-1.638 - -1.308) |
| High-income North America* | 0.877 (0-1.895) | 0.267 (0-0.578) | 0.814 (0-1.740) | 0.126 (0-0.268) | -2.399 (-2.569 - -2.228) |
| Tropical Latin America | 0.078 (0.028-0.126) | 0.078 (0.028-0.126) | 0.180 (0.065-0.294) | 0.068 (0.025-0.111) | -0.461 (-0.660 - -0.262) |
| Caribbean | 0.023 (0.008-0.039) | 0.087 (0.028-0.148) | 0.050 (0.018-0.083) | 0.093 (0.034-0.154) | 0.261 (0.098-0.425) |
| Central Latin America | 0.062 (0.023-0.102) | 0.071 (0.026-0.116) | 0.129 (0.048-0.217) | 0.050 (0.019-0.084) | -1.249 (-1.436 - -1.062) |
| Oceania | 0.001 (0.000-0.002) | 0.033 (0.011-0.066) | 0.004 (0.001-0.007) | 0.041 (0.014-0.077) | 0.716 (0.403-1.030) |
| Central Europe* | 0.295 (0-0.636) | 0.189 (0-0.409) | 0.383 (0-0.817) | 0.183 (0-0.390) | -0.134 (-0.352-0.085) |
| Andean Latin America | 0.014 (0.005-0.022) | 0.064 (0.025-0.105) | 0.035 (0.014-0.060) | 0.058 (0.023-0.100) | -0.126 (-0.943-0.697) |
| Eastern Europe* | 0.455 (0-0.969) | 0.152 (0-0.324) | 0.236 (0-0.523) | 0.068 (0-0.151) | -2.460 (-3.036 - -1.881) |
| Southeast Asia | 0.227 (0.086-0.372) | 0.079 (0.030-0.129) | 0.769 (0.284-1.275) | 0.104 (0.039-0.173) | 0.863 (0.796-0.931) |
| South Asia | 0.159 (0.054-0.263) | 0.024 (0.008-0.039) | 0.445 (0.164-0.731) | 0.028 (0.010-0.045) | 0.510 (0.355-0.665) |
| East Asia | 1.619 (0.604-2.723) | 0.165 (0.062-0.278) | 5.371 (1.930-9.044) | 0.229 (0.082-0.385) | 1.067 (0.794-1.341) |
| North Africa and Middle East | 0.154 (0.059-0.258) | 0.080 (0.030-0.134) | 0.312 (0.118-0.506) | 0.062 (0.023-0.100) | -0.837 (-1.058 - -0.615) |
| Southern Sub-Saharan Africa | 0.018 (0.007-0.029) | 0.059 (0.022-0.097) | 0.018 (0.007-0.030) | 0.028 (0.010-0.047) | -2.505 (-3.081 - -1.926) |
| Eastern Sub-Saharan Africa | 0.019 (0.007-0.033) | 0.022 (0.008-0.038) | 0.047 (0.016-0.081) | 0.024 (0.008-0.042) | 0.238 (-0.006-0.483) |
| Western Sub-Saharan Africa | 0.010 (0.003-0.016) | 0.010 (0.004-0.016) | 0.024 (0.008-0.042) | 0.011 (0.004-0.019) | 0.300 (0.300-0.300) |
| Central Sub-Saharan Africa | 0.008 (0.003-0.015) | 0.030 (0.011-0.057) | 0.016 (0.005-0.033) | 0.024 (0.008-0.047) | -0.787 (-0.998 - -0.575) |
| Notes: AAPC, average annual percentage change; CI, confidence interval; UI, uncertainty interval; SDI, sociodemographic index; *Any uncertainty intervals with bounds outside the plausible range of the indicator were truncated at the theoretical minimum (0) or maximum possible value for interpretability. | | | | | |
