# Supplementary figures and images for: Cancer burden and health inequalities attributable to occupational arsenic exposure: A 32-year global, regional, and national observational study with projections to 2036
Source: Medicine (Baltimore). 2026 Jul 31;105(31):e49979. doi: 10.1097/MD.0000000000049979 (PMC13433024; doi:10.1097/MD.0000000000049979)

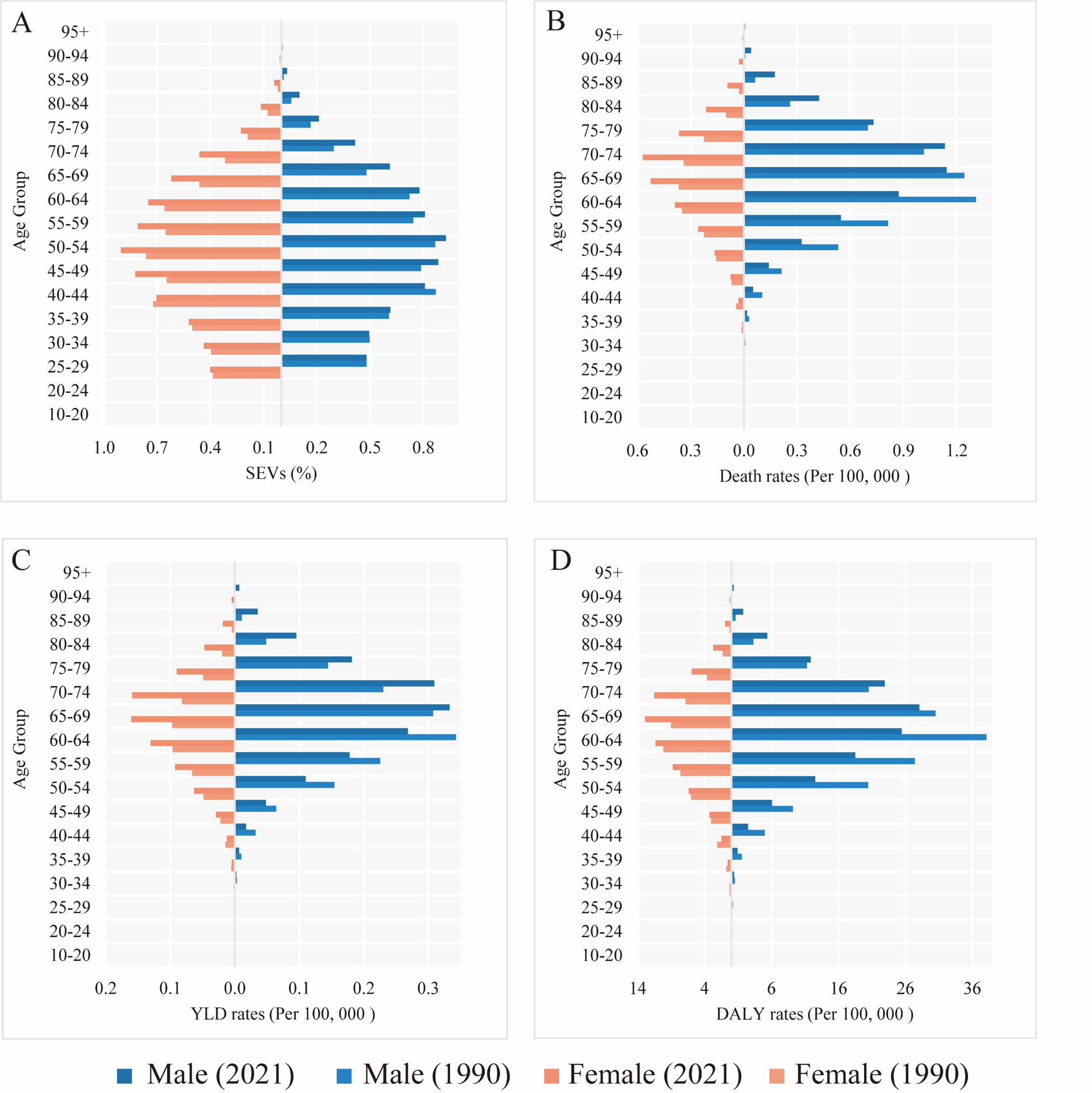

Supplement: Supplementary file 3 [file medi-105-e49979-s003.tif]

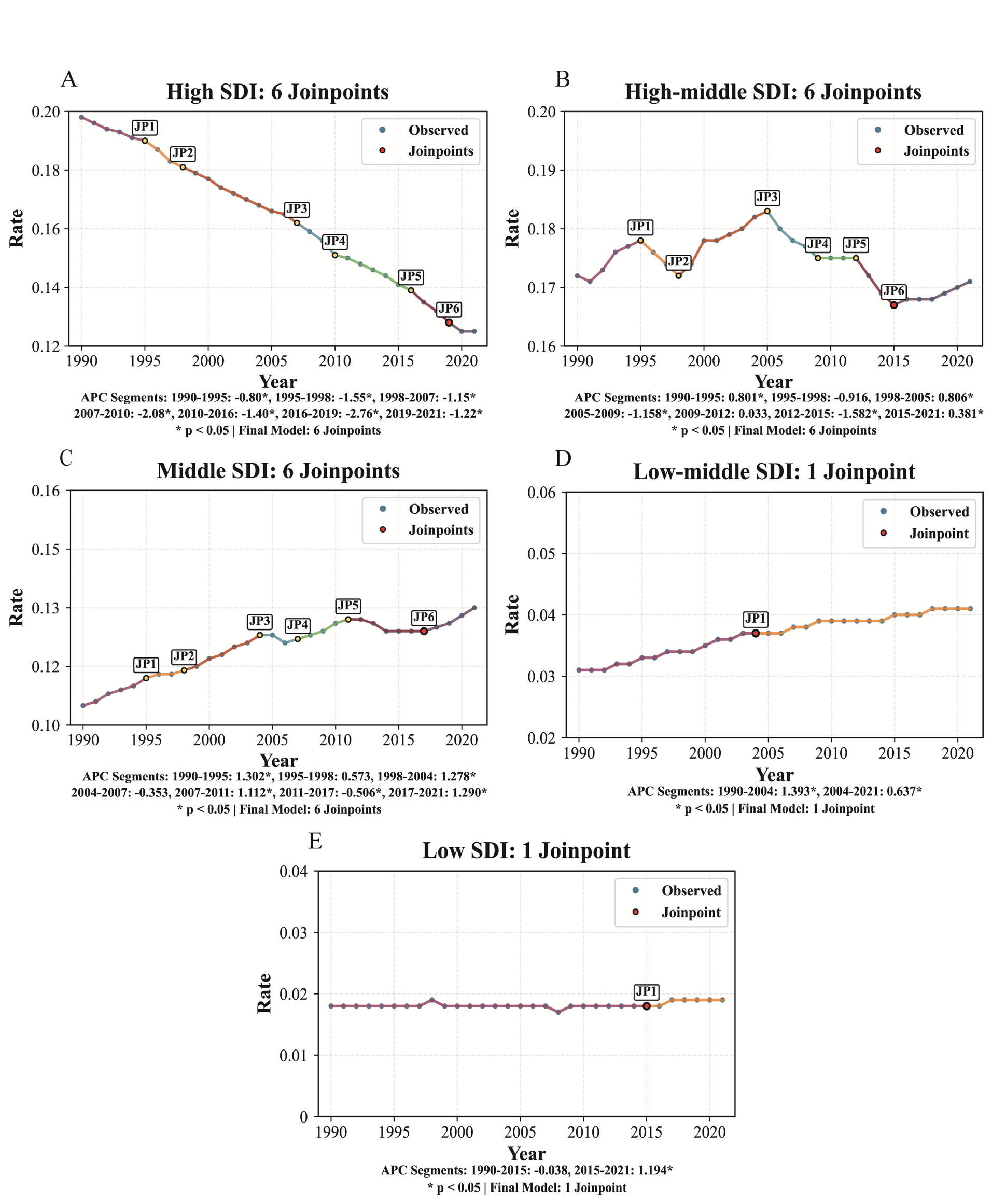

Supplement: Supplementary file 4 [file medi-105-e49979-s004.tif]

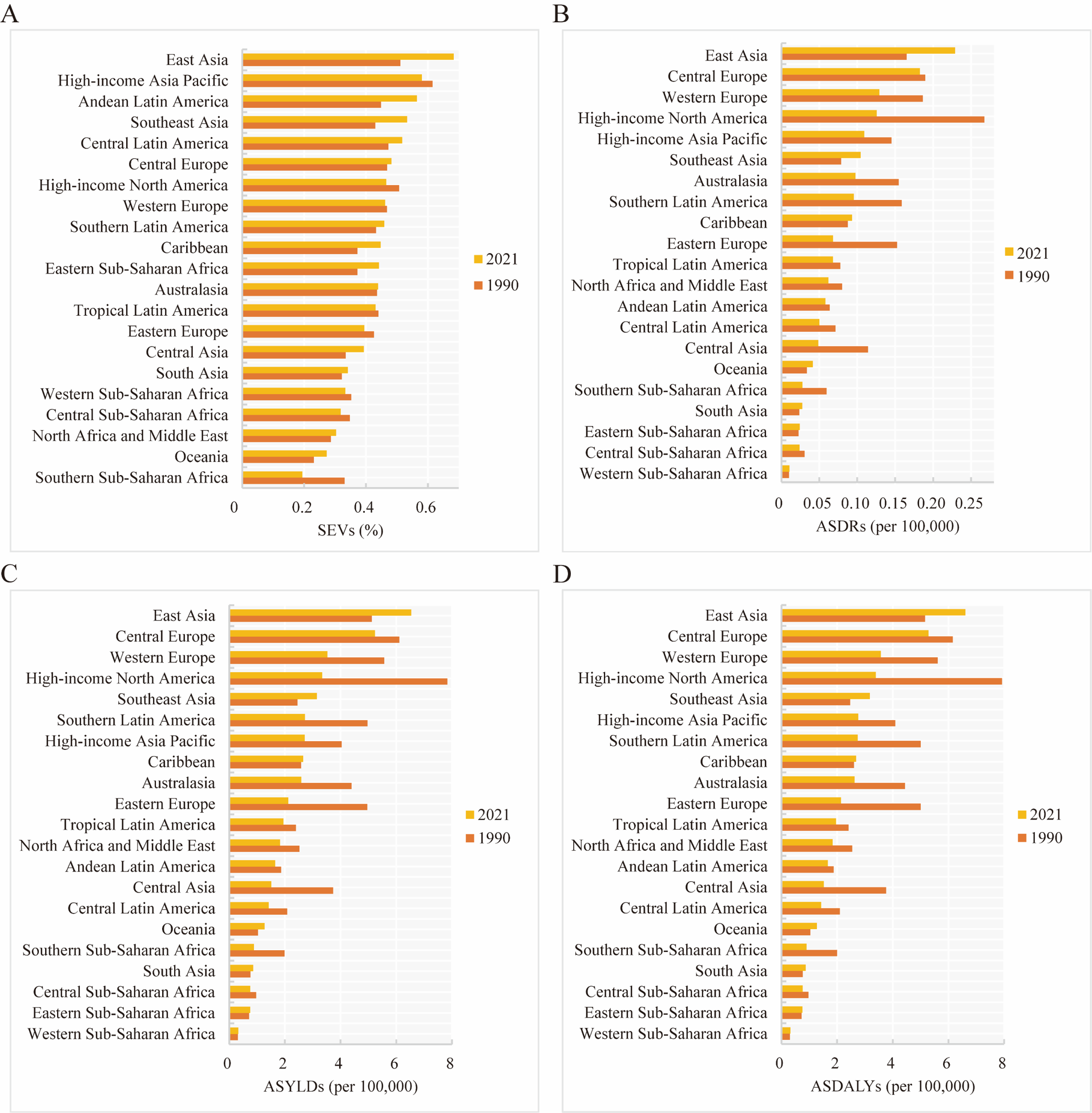

Supplement: Supplementary file 5 [file medi-105-e49979-s005.tif]

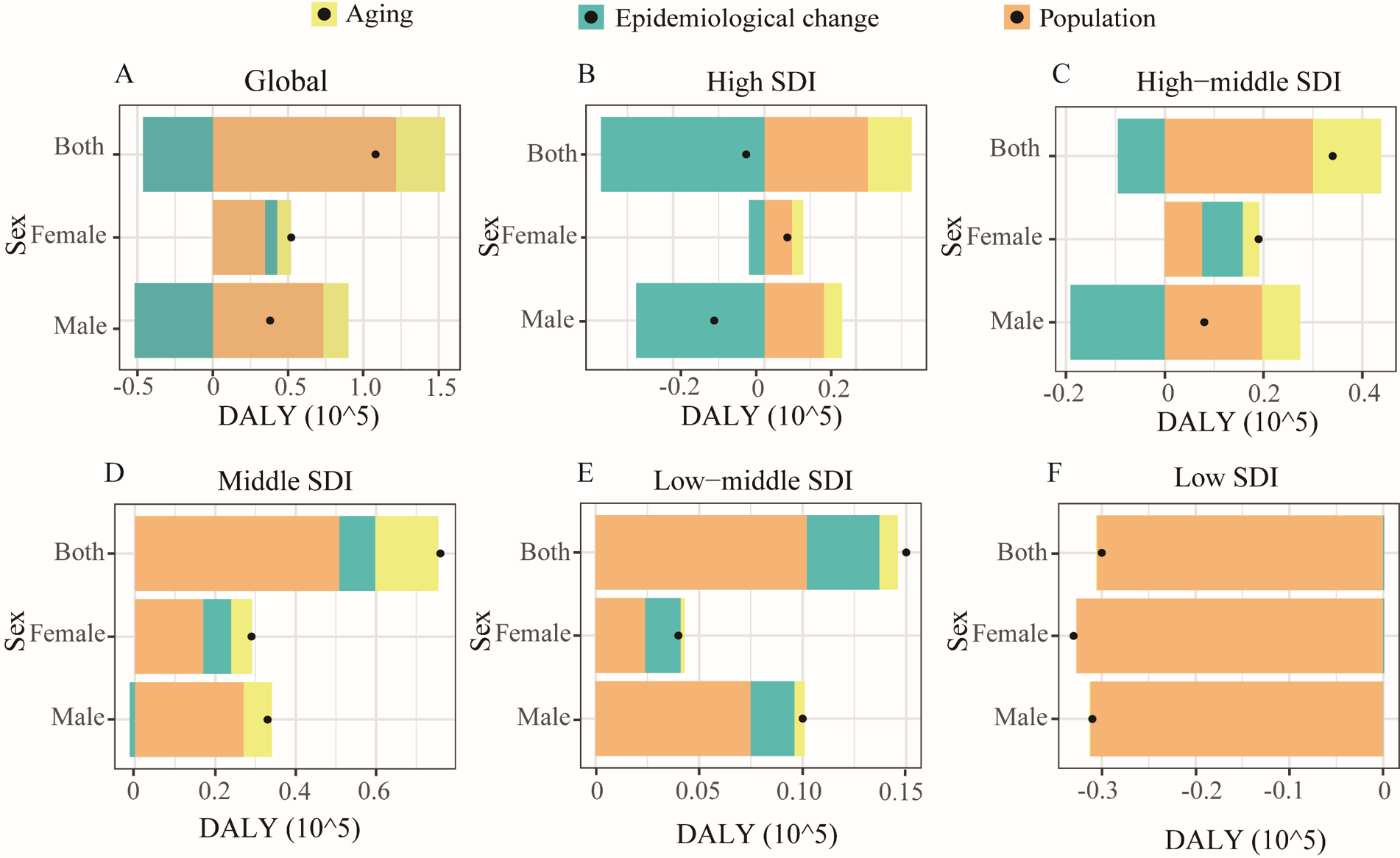

Supplement: Supplementary file 6 [file medi-105-e49979-s006.tif]

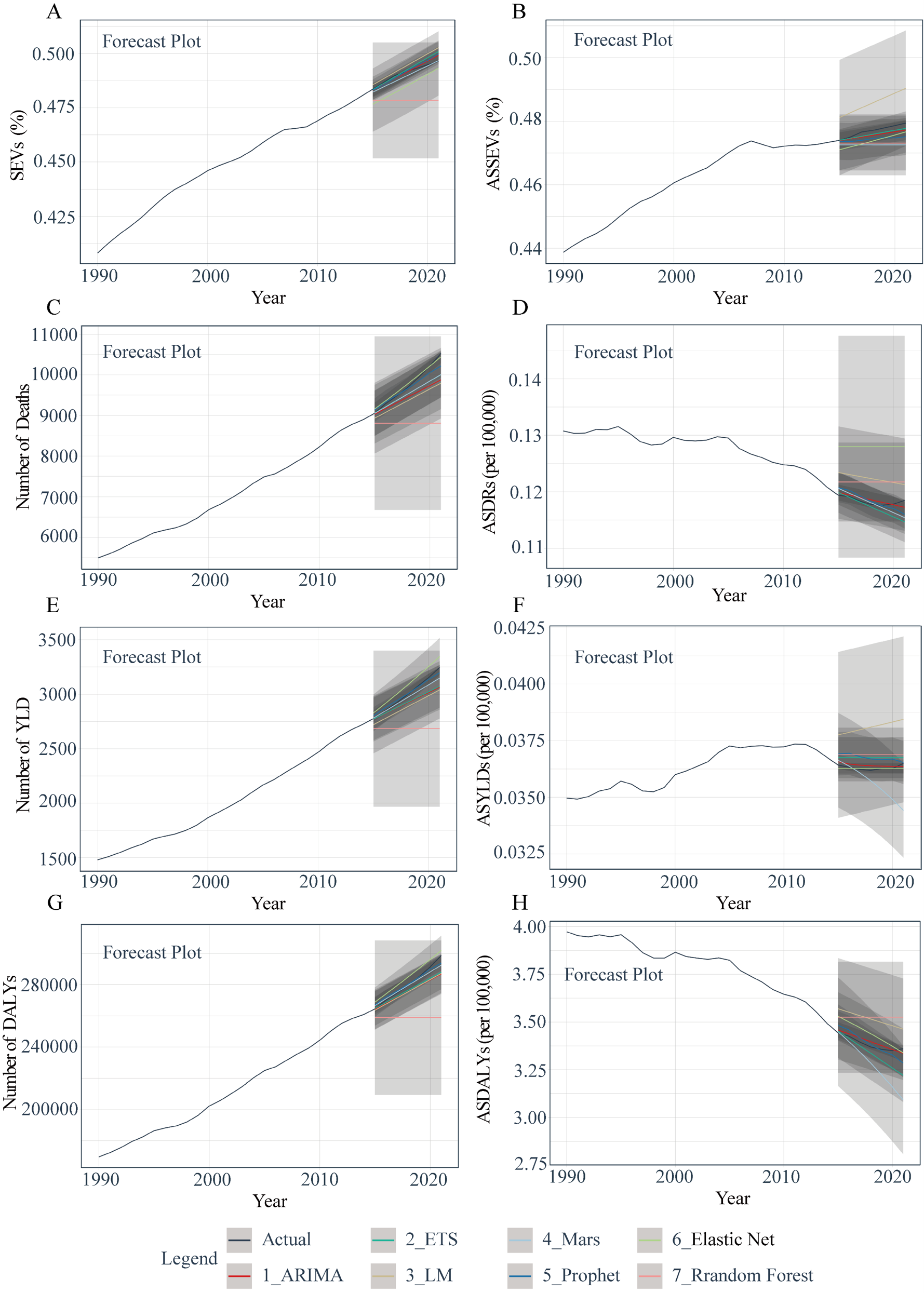

Supplement: Supplementary file 7 [file medi-105-e49979-s007.tif]
